# Smart Healthcare at Home: A Review of AI-Enabled Wearables and Diagnostics Through the Lens of the Pi-CON Methodology

**DOI:** 10.3390/s25196067

**Published:** 2025-10-02

**Authors:** Steffen Baumann, Richard T. Stone, Esraa Abdelall

**Affiliations:** 1 Department of Industrial and Manufacturing Systems Engineering, Iowa State University, Ames, IA 50011, USA; rstone@iastate.edu; 2 Industrial Engineering Department, Jordan University of Science and Technology, Al Ramtha 22110, Jordan; abdelallesra@gmail.com

**Keywords:** AI-enabled healthcare, IoT-enabled devices, wearables, body composition, Pi-CON methodology, usability, remote patient monitoring, virtual care, AI, FDA

## Abstract

The rapid growth of AI-enabled medical wearables and home-based diagnostic devices has opened new pathways for preventive care, chronic disease management and user-driven health insights. Despite significant technological progress, many solutions face adoption hurdles, often due to usability challenges, episodic measurements and poor alignment with daily life. This review surveys the current landscape of at-home healthcare technologies, including wearable vital sign monitors, digital diagnostics and body composition assessment tools. We synthesize insights from the existing literature for this narrative review, highlighting strengths and limitations in sensing accuracy, user experience and integration into daily health routines. Special attention is given to the role of AI in enabling real-time insights, adaptive feedback and predictive monitoring across these devices. To examine persistent adoption challenges from a user-centered perspective, we reflect on the Pi-CON methodology, a conceptual framework previously introduced to stimulate discussion around passive, non-contact, and continuous data acquisition. While Pi-CON is highlighted as a representative methodology, recent external studies in multimodal sensing, RFID-based monitoring, and wearable–ambient integration confirm the broader feasibility of unobtrusive, passive, and continuous health monitoring in real-world environments. We conclude with strategic recommendations to guide the development of more accessible, intelligent and user-aligned smart healthcare solutions.

## 1. Introduction

### 1.1. The Shift Toward Smart Healthcare at Home

The convergence of an aging global population, rising chronic disease burden and post-pandemic transformations in care delivery have accelerated the demand for home-based medical technologies. In the United States, the proportion of individuals aged 65 and older is expected to exceed 20% by 2030 [1]. Globally, health systems face similar challenges as life expectancies increase and multimorbidity becomes more common, placing additional strain on hospital-based care [2].

The COVID-19 pandemic served as a major accelerator for this shift. In response to public health restrictions and hospital capacity concerns during the pandemic, healthcare providers rapidly adopted telehealth and remote patient monitoring (RPM) solutions [3]. In parallel, regulatory bodies such as the Centers for Medicare & Medicaid Services (CMS) expanded reimbursement coverage and introduced new CPT (Current Procedure Technology) codes to incentivize RPM services [4]. These shifts laid the groundwork for broader acceptance and integration of home-based digital health technologies. This transformation was enabled by rapid advancements in AI, sensor miniaturization, and non-contact monitoring capabilities, making smart healthcare at home feasible, scalable, and personalized [5]. In this review, the Pi-CON framework is used as a conceptual lens to structure discussion of passive, non-contact, and continuous health monitoring, serving as a representative example within a wider range of unobtrusive and ubiquitous multimodal frameworks that are emerging.

### 1.2. Growth of AI-Enabled Wearables and Diagnostics

AI-enabled medical wearables and home-based diagnostics are now central to the evolving digital health ecosystem. The global market for AI-powered wearables is forecasted to surpass USD 39 billion by 2026, driven by demand for real-time data, self-management tools, and chronic care support [6]. In the U.S. alone, wearable device usage rose sharply during the pandemic, with 44.5% of adults reporting use of at least one wearable health tracker within the next year [7].

The capabilities of these devices have evolved rapidly. Modern wearables can monitor heart rate, respiratory rate, oxygen saturation, and sleep, often in real time and with AI-driven feedback. Similarly, smartphone-based diagnostics are emerging for skin cancer screening, cardiovascular health assessments, and infectious disease testing, making use of a smartphone’s native capabilities. Many of these tools rely on embedded AI models for signal interpretation, anomaly detection, or personalized insights [8].

Another important category of home-based technologies includes body composition assessment tools, which are increasingly used to track fat mass, muscle mass, bone content, water content, and metabolic trends. While traditional body composition analysis requires clinical-grade tools like DEXA, or sometimes referred to as DXA (Dual-Energy X-ray Absorptiometry), new solutions, such as smart scales and smartphone camera-based assessments, are making this information more accessible for at-home users [9]. These technologies enable users to monitor health trends longitudinally, offering insights that go beyond weight alone, and are beginning to support applications in fitness, metabolic risk tracking, and even early detection of sarcopenia in older adults [10].

### 1.3. Usability and Adherence: A Growing Concern

Despite their promise, many smart healthcare tools, however, face challenges with long-term engagement and usability. Studies show that a significant proportion of users discontinue wearable device use within months, often due to complexity, discomfort, technical issues, or perceived lack of value [11]. For older users in particular, physical limitations and digital literacy barriers can undermine consistent use and compromise health outcomes [12].

As a matter of fact, a 2024 study evaluating wearable adherence among seniors found that more than 30% of participants failed to meet usage expectations during a two-week trial period, reporting difficulty with device setup and discomfort during use [13]. These findings highlight a critical gap: while technology exists, its real-world effectiveness is often limited by human factors and proper usability, not hardware itself.

### 1.4. The Role of AI in Passive, Continuous, Non-Contact Monitoring

AI plays a key role in enabling tools that reduce user burden. It is increasingly applied to power passive sensing systems, such as camera-based photoplethysmography (PPG), radar-based vitals monitoring, and ambient sensors that do not require physical contact with the user [14]. These systems offer unobtrusive, real-time data collection with minimal user interaction, making them especially suited for aging populations and individuals with chronic illnesses.

Beyond sensing, AI algorithms can analyze large volumes of physiological data, detect early signs of deterioration, and deliver personalized feedback, all without relying on patients to manually input data or interpret complex outputs [15]. This automation is essential for scaling smart healthcare across diverse populations and home environments.

A key dimension highlighted in this review is the distinction between IoT (Internet of Things) and AI in wearable healthcare systems. While IoT architecture primarily addresses connectivity, interoperability and transmission of physiological data across devices, AI provides the computational intelligence required to interpret and transform these raw signals into clinically meaningful insights. For this review, the emphasis is placed on AI methods most relevant to passive, non-contact and continuous monitoring. These include machine learning classifiers and signal-processing algorithms for noise reduction and artifact handling, as well as emerging paradigms such as federated learning that enable personalization while preserving privacy. This focus was chosen because such methods support unobtrusive, real-world signal capture at scale, reduce patient burden, and ensure robustness in uncontrolled environments outside a clinic. Clarifying this distinction underscores that IoT provides the backbone for connectivity, while AI systems enable intelligent interpretation and adaptation, together forming the foundation for wearable solutions that are both scalable and clinically meaningful.

### 1.5. Introducing the Pi-CON Methodology

To evaluate the usability and adoption potential of emerging home-based health technologies, this review applies the Pi-CON methodology, a human-centered framework for assessing passive, non-contact and continuous monitoring systems with minimal user burden for data collection. The Pi-CON methodology was first introduced as a conceptual framework for remote patient monitoring usability in virtual health [5]. It was subsequently formalized into a structured usability impact model for smart healthcare technologies [16] and later validated in a controlled study assessing predictive value for user acceptance [17]. These works, together with its adoption by other researchers, establish Pi-CON as a practical approach for evaluating the real-world integration of AI-enabled, home-based health monitoring solutions.

Pi-CON is best understood as a structured evaluation framework. It provides guidance for researchers and developers to examine how health technologies address adoption, usability and patient compliance challenges. In this way, Pi-CON clarifies the process of designing and assessing systems intended to deliver unobtrusive, passive, non-contact and continuous health monitoring in virtual health contexts.

Originally introduced in the context of RPM, Pi-CON emphasizes reducing the cognitive, physical, and behavioral demands (such as avoiding common user errors) while enabling continuous, high-quality physiological data acquisition. By focusing on real-world integration, Pi-CON supports the evaluation of whether these technologies can be sustained in everyday use at home, offering insights into usability, adoption and long-term value in health management.

Unlike conventional device classifications focused on sensor type or platform, Pi-CON centers on the user experience. Systems aligned with this methodology require no wearables, no manual input and can operate unobtrusively in the background of the user’s daily life. The Pi-CON framework offers a valuable lens to assess where current AI-enabled home technologies succeed and where they fall short in supporting accessible, intelligent, and sustainable healthcare at home.

The Pi-CON framework offers a valuable lens to assess where current approaches succeed or face challenges in home health. To support this, initial comparative testing of a Pi-CON–based non-contact vital-signs sensor versus conventional patient-generated health data (PGHD) devices found that participants preferred the non-contact acquisition workflow and interface, with fewer operator errors (0.33 Vs. 0.85 errors per measurement) [17]. This review applies the Pi-CON lens to analyze recent developments in wearables, digital diagnostics, and body composition assessment tools. We focus on technical performance, real-world usability, and the degree to which these systems meet the promise of seamless integration into patients’ lives.

## 2. Review Methodology

This paper presents a narrative literature review that synthesizes current knowledge across AI-powered health monitoring technologies. A structured literature search was conducted using PubMed, IEEE Xplore, ScienceDirect and Google Scholar for studies published primarily between 2020 and 2025. Search terms included “AI in healthcare,” “wearable diagnostics,” “non-contact sensors,” “continuous monitoring,” and “user engagement in digital health.” Older references were selectively included when foundational to the topic (such as regulatory standards or long-standing usability frameworks). Studies were chosen based on relevance, quality and originality. To ensure transparency and quality, the SANRA (Scale for the Assessment of Narrative Review Articles) criteria were followed throughout the review process.

## 3. Overview of Home-Based Health Technologies

### 3.1. Vital Sign Monitoring Wearables

Vital sign monitoring is a foundational use case for home-based health technologies, with smartwatches and wrist-worn devices among the most common form factors. These devices typically track heart rate, heart rate variability (HRV), blood oxygen saturation (SpO_2_), respiratory rate, skin temperature, and activity levels by using a combination of optical, motion and thermal sensors. Advanced models also incorporate algorithms for atrial fibrillation detection, sleep staging, and stress estimation [18].

Cuffless blood pressure monitors are also emerging as a practical tool for continuous hypertension management at home. While traditional devices remain the clinical standard, newer PPG-based and pulse transit time (PTT)-based systems are under active development and early regulatory review [19].

The accuracy of consumer-grade devices varies widely depending on the desired application, device placement, activity level, and population. Other factors that affect the accuracy include poor fit, motion artifacts, and skin tone variation, particularly with optical based sensors. A 2023 umbrella review found that heart rate measurements are generally accurate under resting conditions, while SpO_2_ and respiratory rate are more variable [20]. Regulatory clearance also varies: some devices require FDA clearance for specific features (such as for pulse oximetry), while others remain in the wellness category, with limited clinical validation available. This variability raises concerns about overreliance on devices that may not be fit for diagnostic use [21].

Despite these challenges, wearable monitoring continues to grow in popularity due to its convenience, real-time feedback and integration with mobile platforms. AI models play a central role in making sense of continuous signals, filtering motion artifacts, and translating raw data into personalized insights [22]. In other words, the use of AI models enabled the detection of vitals deviations that signify potential health issues, facilitating early warning and better health management for patients and healthcare provider.

### 3.2. Digital Diagnostics and At-Home Testing Tools

Alongside wearables, digital diagnostics are redefining how users access and interpret health information at home. These tools encompass a range of self-guided, AI-enabled tests that use smartphone cameras, sensors, or connected devices to screen for medical conditions.

One prominent category includes smartphone-based electrocardiogram (ECG) systems which enable users to capture a single-lead ECG and receive immediate analysis. These tools have demonstrated strong clinical utility for atrial fibrillation (Afib) detection, particularly in older adults and patients with known cardiovascular risk [23]. Similarly, AI-powered dermatology apps allow users to photograph skin lesions and receive classification risk scores based on trained image recognition models. While some of these tools have demonstrated accuracy comparable to that of dermatologists in controlled studies, real-world performance is more variable due to lighting, image quality, and user errors [24].

The COVID-19 pandemic also paved way for the innovation in self-administered testing kits, ranging from antigen swabs to connected digital readers for multiplex pathogen detection. Many of these tools now integrate with mobile apps for guided use and results interpretation, increasing accessibility for non-clinical users [25].

A common thread among these tools is reliance on AI for data interpretation, signal classification and user guidance [22]. This shift represents a decentralization of care with opportunities and challenges, particularly regarding user training, false positives, and seamless follow-up with healthcare providers. Ensuring clear instructions, regulatory oversight, and robust data-sharing mechanisms remain essential as these technologies mature [26].

### 3.3. Body Composition Assessment Technologies

Body composition assessments have traditionally relied on clinical tools like DEXA and bioelectrical impedance analysis (BIA) systems [27]. In recent years, a growing range of home-based body composition tools have emerged, enabling users to estimate fat mass, lean mass, and other metrics with varying accuracy and usability. Common tools to estimate these parameters are smartphone apps and smart scales, which integrate BIA technology. While convenient and app-connected, these scales are sensitive to hydration levels, foot positioning, and environmental conditions, which can affect consistency and clinical reliability [28].

Smartphone-based body scanning applications, such as camera–enabled body scanning apps, offer a solution to estimate a user’s body composition and changes in body, circumference and volume. These systems enable the passive tracking of health and fitness trends without requiring users to step on a scale or wear a device, while AI compensates for lighting, pose variation, and segmentation, but studies show mixed results compared to gold-standard methods [29].

One leading example is Spren [30], a company that transforms a smartphone camera into a validated body composition lab. A study reports high concordance with DEXA scans (r ≈ 0.96) and mean absolute error of ~2.3% on 5500+ users across demographics [30]. The company’s app estimates fat and lean mass, metabolic health, and HRV, all using a camera scan and cloud-based AI processing.

While many of these tools are not yet approved for diagnostic use, their ability to engage users in long-term tracking offers significant promise for preventive care. Ongoing validation, usability testing, and feedback integration will be critical to broaden clinical acceptance.

Furthermore, Starkoff & Nickerson [31] conducted a narrative review examining the emergence of mobile health (mHealth) tools for at-home body composition assessment, centered on ultrasound, DEXA and AI-powered imaging, supported by telehealth platforms. The results show that DEXA and ultrasound in clinical settings remain gold-standard methods, as DEXA excels at quantifying bone mineral density, fat mass, and lean mass, while ultrasound offers practical, low-cost measurement of subcutaneous fat and muscle thickness. The authors also spotlight AI-enhanced image analysis and mHealth apps that leverage smartphones, computer vision, and deep learning. These tools aim to replicate clinical-grade assessments in home environments. Such innovations provide real-time feedback, personalized metabolic monitoring, and individualized guidance, but their validity, reliability, and inclusivity still need thorough validation. The authors’ review emphasizes that bridging technological capability with clinical accuracy and ensuring equitable application across different populations are essential for transitioning these tools from pilot to mainstream use.

Even with the significant promises AI offers, its accuracy relies on the quality and representativeness of the datasets used for training. A study by Lee et al. [32] demonstrated that an AI model designed for body composition analysis exhibited bias, leading to a significant decrease in outcome accuracy when imbalanced training datasets were utilized. Specifically, the study found that white individuals were overrepresented in their trained image dataset. This overrepresentation resulted in poorer accuracy for black participants, which is attributed to distributional differences within the image data.

## 4. Previous Work

### 4.1. Usability, Engagement, and Barriers to Adoption

While AI-enabled health technologies have rapidly expanded in capability, real-world adoption continues to lag their clinical and technical promise. A recent scoping review noted that many AI solutions face adoption barriers due to implementation challenges, poor integration into user workflows and limited end-user acceptance [33]. The core challenge lies not only in sensor accuracy or algorithmic performance but also in the user experience, ensuring the provided device is both safe and intuitive to use. Usability, defined by ISO 9241-11 as “the extent to which a system, product or service can be used by specified users to achieve specified goals with effectiveness, efficiency and satisfaction in a specified context of use” [34], remains a decisive factor in adoption outcomes. Similarly, long-term engagement depends on whether users perceive ongoing value, understand provided results, and can easily incorporate the device into existing routines.

Even well-performing systems face limited usage if setting up a device, for instance, if it is unclear or if the interface excludes individuals with limited technical literacy or physical abilities. Recent research has emphasized the need for simple-to-understand user interfaces (UI) and instructions in AI-driven health feedback, especially as these tools are deployed across increasingly diverse populations [35].

Recent studies highlight the value of personalization in improving user engagement with wearable devices. Li and Washington [36] found that personalized machine learning models (those trained on data from individual users rather than the general population) significantly outperformed generalized models. When combined with tailored onboarding, these systems fostered greater trust and long-term use. Similarly, Wang [37] demonstrated that fatigue-aware interfaces, which adjust in real time based on physiological feedback like eye strain or heart rate variability, improved usability ratings by 22% among older adults.

These findings underscore the need for AI-driven, adaptive systems that lower the cognitive burden of onboarding and improve usability from the outset.

To explore these challenges in depth, this review section is organized into six core themes:

(1) user interface design and onboarding;
(2) the novelty effect and sustained engagement;
(3) usability across populations;
(4) AI applications in home-based monitoring;
(5) trust, privacy, and data security.

Each subsection draws on recent literature to highlight both progress and persistent gaps in the field.

### 4.2. User Interface Design and Onboarding

One of the most critical stages in the adoption of home-based health technologies is the onboarding process, meaning the user’s initial experience when setting up and learning how to use a device, app, or system for the first time. In this context, onboarding refers to several key steps such as powering the device on, connecting to an app, pairing with Bluetooth Wi-Fi, creating an account, entering personal info, and initial calibration or personalization [38].

The authors also state that the onboarding process remains a pivotal determinant of long-term device adoption. Early interactions, including where users setup, pair and begin engaging with a health device, can make or break usage continuity. Research illustrates that older adults and individuals with limited digital literacy are particularly susceptible to abandonment during this phase, underscoring the importance of thoughtful, user-centric onboarding strategies [39].

Hou [40] reports that older adult users often struggle with technical jargon and unclear setup instructions for wearable devices, frequently leading them to abandon the process prematurely. And Denecke et al. [41] emphasize that the effectiveness of digital health interventions hinges on careful consideration of health literacy, advocating for user-centered design and simplified language to ensure accessibility for all. Together, these findings highlight the critical need for intuitive design and clear communication in digital health tools to bridge the usability gap, particularly for older adults and individuals with varying levels of health literacy. Addressing these challenges is essential for maximizing the adoption and health benefits of digital technologies.

Physical comfort during onboarding may impact the user experience. Jan et al. [42] reported that around 14% of wearables users experienced skin irritation,. While wrist-worn devices are generally designed without adhesives, irritation may occur from straps or bands. In contrast, devices intended for continuous or specialized monitoring (such as continuous glucose monitors) often require adhesives, where irritation rates as high as 35% have been reported [43]. Research has consistently shown that a confusing onboarding process leads to frustration and high early abandonment rates. In their deployment study of standalone voice assistants, Chen, Ding et al., 2023; Chen, Lifset et al. [2,44] observed that older adults often struggled with tasks like device pairing and activation due to ambiguous prompts and unfamiliar interaction patterns. The authors reported a significant improvement in success rates when multimodal support (such as screen prompts, voice guidance and tactile feedback) was provided directly on the device. This underlines the importance of accessible design features like voice assistance, tactile indicators and high-contrast visuals for increasing inclusivity in home-based health monitoring.

Similarly, Shandilya & Fan [45] found that many older adults expressed interest in AI-enabled IoT healthcare tools, yet their experiences were hampered by anxiety about privacy, loss of control and insufficient instruction during setup. Transparency in the onboarding experience, such as clear communication about how data will be used and which sensors are active was cited as a key determinant of trust.

To address these challenges, recent approaches have adopted AI-driven adaptive onboarding flows that personalize tutorials based on user behavior. Evans & Agoro [46] demonstrated that embedding generative AI into onboarding interfaces allowed real-time adaptation to user responses and simplified setup experiences. Their study tracked users across three age groups during device setup. They measured “time to successful activation” (the duration from unboxing to first successful data transmission) and collected satisfaction ratings via post-onboarding surveys using a Likert scale. Participants who received natural-language guidance activated devices faster and scored higher on overall satisfaction, compared to those following standard instruction manuals.

Supporting this, Costa et al. [47] found that incorporating adaptive gamification into onboarding workflows increased early engagement and reduced dropout. Their AI-based system adjusted tutorial pacing and reward mechanisms in real-time, based on the user’s behavioral patterns. Similarly, Lu et al. [48] reported that personalized onboarding supported by motivational feedback improved adherence in users managing chronic illness, as users felt more in control and better understood the setup process.

In conventional wearables, such gamification is typically delivered through web- or app-based user interfaces, where design choices play a critical role. UI design plays an important role as well. Sandhaus et al. [49] emphasized that interfaces employing progressive disclosure where information is revealed step by step rather than all at once, with the results of a reduced cognitive load and increased trust during onboarding. Their study on in-vehicle health monitoring showed that clear, contextual cues helped users understand how data was collected and used, which improved adoption rates even among skeptical users.

Overall, these findings suggest that onboarding is not a one-time hurdle but a formative moment in the user journey that must be intentionally designed. Successful onboarding in AI-powered smart health technologies should be intuitive, context-sensitive and supportive across varying levels of literacy, ability and familiarity with technology. As more devices leverage AI and IoT to deliver personalized healthcare at home, ensuring a seamless onboarding experience will be essential to building trust, improving usability and fostering long-term engagement.

**Figure 1:**
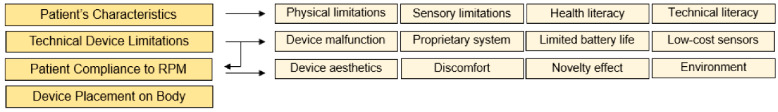
Elements of the RPM usability impact model.

To better understand and quantify how these usability factors affect the adoption and sustained use of RPM systems, the RPM usability impact model [16], as shown in Figure 1, offers a structured framework.

This model outlines how user interface design, onboarding clarity, accessibility and feedback mechanisms influence not only initial engagement but also long-term adherence, data quality, and clinical integration with integrated elements. In this review, Figure 1 is intended as a summary of common barriers encountered with wearable sensors, providing the context for why alternative frameworks such as Pi-CON are being explored. It provides a practical tool for evaluating how usability translates into measurable health and business outcomes, making it especially relevant in scaling AI-IoT health technologies for diverse populations.

### 4.3. Overcoming the Novelty Effect in AI-IoT Health Systems

A well-documented challenge in the adoption of wearable and home-based health technologies is the novelty effect, a phenomenon where users display high initial enthusiasm that rapidly diminishes over time [50]. In the context of smart healthcare, this early excitement often stems from the promise of new insights, sleek design or gamified features, but it fades as the device becomes less engaging or fails to integrate meaningfully into daily routines.

Shin et al. [51] conducted a longitudinal study on wearable activity trackers and found that most users exhibited a “novelty peak” during the first two to four weeks of use, followed by a noticeable drop in engagement. Interestingly, some users re-engaged with the device after an initial decline, often due to reminders, new insights or health changes that made the device relevant again. This pattern underscores that while novelty can drive adoption, sustained use depends on deeper behavioral integration.

The concept of value co-creation also emerged as a critical factor. Windasari & Lin [52] found that long-term wearable use is strongly associated with whether users perceive the device as contributing to their ongoing health goals. When wearables offer real-time feedback, integrate with broader health ecosystems or provide access to personalized insights, users are more likely to develop a sense of ownership and maintain usage.

Gamification is another widely adopted but inconsistently effective strategy. Hydari et al. [53] examined Fitbit’s leaderboard feature and reported that it initially increased daily step counts by approximately 370 steps; however, the effect was short-lived unless gamified elements were continually updated and personalized. This aligns with findings from Dhiman [54], who emphasized that static challenges and rewards decrease quickly. To be effective, gamification must evolve in tandem with user progress and incorporate real-time AI personalization, haptic nudges and social accountability features such as family leaderboards or clinician-linked encouragement [55].

Recent work by Alsaad et al. [56] supports this need for personalization by demonstrating how multimodal large language models (MLLMs) can sustain engagement through conversational interfaces that evolve with the user’s needs over time. These MLLMs adapt in context, using sensor input and behavioral patterns to fine-tune prompts, thereby reinforcing motivation and maintaining relevance. Additionally, Kargarandehkordi et al. [57] illustrated that real-time AI models embedded in wearables can predict stress-induced blood pressure spikes, prompting personalized or actionable health nudges. These proactive interactions can help re-engage users during low-interest phases by making the technology feel more responsive and clinically valuable.

The literature makes clear that mitigating the novelty effect is not just about keeping users interested. It is about creating systems that adapt, resonate with personal health journeys, and deliver incremental value over time. The integration of real-time AI responsiveness and multimodal feedback loops may mark a shift from short-term novelty to long-term necessity. AI-enabled personalization, adaptive goal settings, and timely interventions appear to be essential ingredients for long-term engagement.

### 4.4. Ensuring Inclusive Usability in AI Driven IoT Devices

AI-powered IoT healthcare devices hold immense promise for enabling personalized, home-based monitoring, but their real-world success hinges on inclusivity. Usability must extend across a spectrum of user demographics, including older adults, individuals with disabilities and users with limited digital literacy. Design choices that neglect this diversity risk exclusion, early abandonment or misuse of the technology.

A meta-synthesis by Moore et al. [58] reviewed 15 qualitative studies involving older adults and found that wearables were often abandoned due to discomfort, interface complexity and a lack of contextual guidance. Interestingly, it was not the technical functionality that hindered use, but perceived usefulness and difficulty in integrating the device into daily routines. This reinforces the idea that usability is as much about motivation and support as it is about interface design.

Furthermore, Muñoz Esquivel et al. [59] conducted a large survey of older wearable users and confirmed these findings quantitatively. Continued use was strongly linked to clarity of health feedback, font readability and simple navigation. Users expressed frustration with information overload and inconsistent visual hierarchies, leading to disengagement over time.

A systematic review by [60] found that digital health literacy among adults aged 65 and older is strongly influenced by age, education level and socioeconomic status. These are factors that directly impact their ability to adopt and effectively use digital health tools. Similarly, a narrative review by Fakhimi et al. [61], focusing on smart home and wearable technologies for older adults, highlighted frequent usability challenges, including non-intuitive interfaces, limited personalization and a lack of user feedback. These shortcomings can lead to abandonment of devices, even when their technical performance is high.

Adaptable user interfaces can dramatically improve outcomes. Kim et al. [62] tested three interface types: textual, visual and voice among older adults using a mobile health app. The voice interface proved most usable, particularly for participants with vision impairments or low literacy. This supports the integration of multimodal interaction systems, especially speech-enabled tools, in smart health solutions targeting diverse populations.

Vigouroux et al. [63] extended this concept by comparing tactile versus voice controls in home automation systems for users with physical and sensory disabilities. Their study found that while both modalities were usable, they addressed different accessibility needs. The authors concluded that systems should be customizable, offering multiple user interface pathways (such as voice, touch, visual feedback) to meet varying user capabilities and preferences.

Building on these findings, Pedroso & Khera [64] highlighted that AI-enhanced consumer devices can improve cardiovascular screening, but that their success is directly tied to ease of use and accessibility, especially in underserved populations. Their study emphasized the value of personalized AI interfaces that adjust not only for language and visual preferences but also for user confidence and interaction history. Švihrová et al. [65] further argued that reinforcement learning can be applied to digital health interventions to refine onboarding pathways over time based on real-world behavior. By using causal inference techniques (statistical methods that aim to identify cause-and-effect relationships), they demonstrated a scalable method to increase long-term adherence, particularly for users with low initial digital health engagement.

Together, these findings highlight that AI-driven health devices should prioritize multilingual, multimodal and multisensory interfaces. Integrating dynamic personalization and adaptive learning systems can further enhance inclusivity and ensure engagement across all user groups. Inclusive usability is not just a design choice but a prerequisite for broad adoption in the evolving landscape of AI-powered smart healthcare.

### 4.5. AI Applications in IoT-Based Home Health Monitoring

AI is revolutionizing home-based health monitoring, particularly when combined with IoT devices that allow for passive and continuous data acquisition. These systems now extend beyond basic tracking into domains such as arrhythmia detection, body composition estimation and intelligent signal fusion, all with the potential to approach clinical-grade accuracy.

One notable development is the emergence of DeepBeat, a multi-task deep learning model designed for wrist-worn wearables. Trained on over one million simulated and 500,000 real photoplethysmography (PPG) data points, DeepBeat achieved an F1 score of 0.96 in atrial Afib detection. The F1 score is a metric that balances precision (how many selected items are relevant) and recall (how many relevant items are selected), with a perfect F1 score being 1.0. The model also demonstrated a sensitivity of 0.98 (correctly identifying those with Afib) and a specificity of 0.99 (correctly identifying those without Afit) in real-world, ambulatory testing scenarios [66].

Similarly, Shen et al. [67] developed a convolutional neural network (CNN) trained on over 4000 h of wrist-based PPG data collected in every day, uncontrolled conditions, with data being collected outside of a clinical or lab environment, where variables such as user movement, lighting, sweating, ambient temperature and device placement are not tightly regulated. Their model achieved an Area Under the Curve (AUC) of 0.95. AUC represents the ability of a model to distinguish between classes, in this case, the presence or absence of Afib, with a value of 1.0 indicating perfect classification.

Expanding on signal quality and robustness, Vo et al. [68] introduced ADSSM (Attention-based Deep State Space Model), a novel architecture that uses attention mechanisms to translate noisy PPG signals into ECG-like waveforms. The resulting model achieved an AUC–PR (Area Under the Precision-Recall Curve) of 0.986 for Afib classification. AUC–PR is a metric particularly useful in cases with imbalanced datasets, as it focuses on the trade-off between precision and recall for the positive class (AFib detection). The result indicates high reliability in identifying Afib even in low-prevalence or noisy conditions, thus closing the performance gap between consumer-grade wearables and clinical-grade ECG systems.

Beyond cardiovascular metrics, AI is also advancing body composition monitoring. Graybeal et al. [69] evaluated a smartphone-based AI system for body composition estimation by using a smartphone against a validated 4-compartment (4C) model, which separates the body into fat mass, water, bone mineral content and residual (primarily protein) mass. This model is considered a gold standard because it accounts for individual variability in tissue densities and hydration, offering greater accuracy than simpler two-compartment models (which only separate fat and fat-free mass). The study involved healthy adults and found that the AI-powered, image-based method achieved a Standard Error of Estimate (SEE) of 2.8% for body fat percentage, meaning its predictions deviated on average by 2.8% from the 4C model. In body composition research, an SEE under 3% is considered acceptable for population-level estimation, especially when using non-clinical tools. The findings support the feasibility of scalable, low-burden AI tools for non-invasive home monitoring.

Building on smartphone-based AI body composition tools, Tinsley et al. [70] evaluated the accuracy and reliability of smartphone-based 3D anthropometric scans, a technique that captures body dimensions, such as circumference and volume, by using 3D imaging instead of manual measurements. The study compared mobile phone apps against two professional-grade infrared scanners. The smartphone app performed 360-degree scans, where users rotate while the phone captures ~150 sequential images. These images are stitched into a full 3D model using non-rigid reconstruction, a method that compensates for minor motion like breathing or shifting posture. To assess precision, the authors calculated Intraclass Correlation Coefficients (ICCs), a statistical measure of repeatability. ICCs for mobile scans reached 0.986 for circumferences and 0.997 for body volume, indicating near-perfect agreement with the professional systems (which ranged from 0.974 to 0.998). The mean absolute error was 0.5 cm (0.9%) for circumferences and 0.8 L (1.1%) for body volume. Additionally, the Technical Error of Measurement (TEM) for mobile scans was under 0.9%, underscoring consistent scan reliability. This study supports the potential of smartphone-based anthropometric scanning as a scalable and accurate alternative to traditional methods. Its findings are directly relevant to AI-IoT applications in home health monitoring.

As discussed in the previous section on inclusive usability (Section 4.4), Pedroso & Khera [64] also contribute to the technical foundation of AI-IoT health monitoring. Their work presented an AI-enhanced cardiovascular platform that integrates heart rate, respiration, and motion signals from consumer wearables. The system achieved early detection of cardiovascular anomalies, such as blood pressure spikes and arrhythmias, by applying federated learning to personalize thresholds based on user context.

In continuity with the behavioral guidance strategies highlighted before, Švihrová et al. [65] demonstrated that reinforcement learning and causal inference models can dynamically guide users toward healthier behaviors based on real-time biometric trends. Their system enables intelligent nudging, such as suggesting personalized goals or timing reminders based on user inactivity or stress levels.

Combined, these developments demonstrate the increasing ability of AI-IoT systems to deliver clinically meaningful health data in a home environment. As the field continues to evolve, emphasis will need to shift toward safeguarding the sensitive data these systems generate. Transparency, robust privacy protocols and user control must accompany algorithmic advancements to lay the groundwork for the next phase: building and maintaining trust in AI-enabled health ecosystems.

### 4.6. Trust, Privacy, and Data Security in AI-IoT Ecosystems

As AI and IoT-enabled health systems grow more integrated into daily care, user trust becomes foundational. The private nature of physiological and behavioral data, combined with often opaque data-sharing policies, has led to significant privacy concerns. According to surveys across the U.S. and Europe, a majority of users express hesitation about wearables and health apps due to fears of unauthorized access, data misuse, and potential surveillance [71]. In addition to privacy risks, recent research has also identified potential mental health harms associated with activity trackers, such as increased anxiety and compulsive behavior related to health tracking, which can further discourage sustained use [72].

Federated learning (FL) has emerged as a promising method to address these concerns while still enabling robust AI model training. FL is a decentralized machine learning approach where data remains on the user’s device, and only model updates, such as gradients or parameters, are shared with a central server. This preserves privacy while enabling learning across a distributed network of devices [73]. Rather than aggregating raw data in centralized servers, FL enables devices to collaboratively train AI models locally, sharing only encrypted updates [74]. This not only reduces risk but aligns with emerging global data protection regulations like General Data Protection Regulation (GDPR), which governs data privacy in the European Union, and the Health Insurance Portability and Accountability Act (HIPAA), which protects health data privacy in the United States [75].

In a recent study, Aminifar et al. [76] introduced a privacy-preserving edge FL framework for wearable and mobile devices, demonstrating how such systems can maintain model performance while avoiding transmission of sensitive health data. This architecture is particularly well-suited for seizure prediction and other time-sensitive diagnoses in resource-constrained mHealth environments. Edge-level computation also supports real-time responsiveness and scalability.

Beyond basic FL, more advanced techniques such as federated ensemble learning and blockchain integration are enhancing both model performance and security. For example, Khan et al. [77] describe a hybrid cloud-based healthcare monitoring system that fuses multiple FL models through ensemble learning while leveraging fog nodes and blockchain for data integrity and secure access control. The system achieved diagnostic accuracy exceeding 99% while preserving privacy and resisting adversarial threats. These approaches align with earlier design frameworks that emphasize transparency and user control in home health monitoring. Baumann et al. [78] proposed that integrating user-centered design with secure data infrastructures, such as blockchain-based distributed ledgers, could increase trust and long-term adherence in wearables and home use medical devices.

Explainability also plays a pivotal role in trust. Users and clinicians are more likely to engage with systems that offer transparent reasoning and control over how data are used or shared. Therefore, privacy-by-design principles must go beyond compliance; they must enable understanding, agency and control [79].

As the complexity of AI-IoT healthcare ecosystems grows, trust must be engineered as a core feature. It must be embedded in architecture and into the algorithm. The recent literature further emphasizes the importance of transparency in data use. Chen & Esmaeilzadeh [80] developed a trust framework featuring real-time data provenance and audit trails, allowing users to track how their data are collected, processed and shared to enhance users’ sense of control and transparency. Similarly, Ref. [81] evaluated the dynamic consent app MyHealth Hub and reported that most participants completed all consent-related tasks successfully, rating the system positively on perceived usefulness and ease of use, underscoring that dynamic, user-managed consent can meaningfully improve user autonomy and trust.

With robust data safeguards in place, the focus shifts from whether users are willing to share their data to how systems maintain transparency and trust throughout their lifecycle.

As Section 4.7 will explore, creating ambient, seamless, and user-centered health monitoring solutions depends on embedding this trust directly into system architecture and user experience design, laying the groundwork for truly ubiquitous AI IoT healthcare. Building on the privacy-by-design approaches, passive and ambient systems offer an additional layer of discretion, operating unobtrusively while minimizing user data input.

The reviewed studies show early signals of a shift toward unobtrusive, non-contact, and continuous health monitoring, particularly through ambient sensing, multimodal diagnostics and AI-enabled interpretation of weak physiological signals. While this review organizes the current landscape, the Pi-CON methodology is introduced in the following sections as one representative lens to reflect on how these trends can be synthesized, compared and advanced toward more usable and integrated solutions.

### 4.7. Toward Passive, Ubiquitous and User-Centered AI-IoT Health Monitoring

It has become increasingly evident that the novelty factor, onboarding challenges and lack of digital literacy with wearables and virtual monitoring devices can lead to abandonment and long-term adherence issues, lessening their adoption rates and potential health benefits. Herein, the integration of AI with IoT technologies aims to unlock the potential for truly ambient and ubiquitous healthcare, meaning that systems fade into the background while continuously delivering insight. However, to ensure adoption and sustained utility, these systems must meet users where they are: in their homes, across diverse circumstances and without demanding extensive interaction and onboarding. This calls for a paradigm shift in how we evaluate and design AI and IoT-enabled health tools.

The Pi-CON methodology, which emphasizes passive, non-contact and continuous monitoring, offers a pragmatic framework to address this shift. Unlike traditional device evaluation frameworks focused on technical specifications or accuracy alone, Pi-CON centers on user burden, seamless integration, and real-world sustainability. The recent literature reinforces the need for this perspective. Pourpanah & Etemad [82] present a comprehensive landscape of in-home health monitoring systems and emphasize the importance of ubiquitous, unobtrusive sensing in delivering value to users. They highlight how non-wearable and ambient sensing approaches, such as radar-based fall detectors and bed-embedded sensors, are gaining traction due to their passive and continuous operation, which aligns with Pi-CON’s foundational goals. These technologies reduce the cognitive and behavioral demands placed on users, making long-term adherence more likely. Similarly, some used ambient audio sensors to track potential respiratory issues, such as chronic coughing and snoring, to facilitate passive health monitoring [83].

Further, Abedi et al. [84] demonstrate the efficacy of a mm-Wave radar system enhanced with AI for continuous gait monitoring in home environments. This solution eliminates the need for wearables or manual input, operating silently and reliably in the background. Such non-contact solutions directly embody Pi-CON’s passive and continuous dimensions while also addressing common usability pain points in older or mobility-impaired populations.

Beyond these case studies, a growing body of work has systematically advanced non-contact sensing across multiple modalities, underscoring its maturity and alignment with Pi-CON principles. Recent advances in non-contact sensing highlight the feasibility of passive, continuous health monitoring across diverse modalities. Millimeter-wave Frequency Modulated Continuous Wave (FMCW) radar systems have been shown to accurately capture heart rate and respiration with up to 93% accuracy, offering unobtrusive monitoring in ambient settings [85]. Similarly, ultra-wideband (UWB) radar techniques demonstrate high robustness across varied postures and environments, achieving heart rate estimation errors as low as 1.32 bpm when benchmarked against ECG [86]. Camera-based methods have also matured, with clinical validation of remote photoplethysmography (rPPG) showing 96% agreement with standard systems for respiratory rate measurement during teleconsultations [87]. Reviews further emphasize the growing strength of deep learning in rPPG applications, addressing motion artifacts and lighting variability to enhance real-world usability [88].

Other innovations include deep learning–driven cuffless blood pressure estimation from PPG signals [89] and multimodal fusion techniques combining rPPG with eyelid dynamics for fatigue detection. The role of non-contact sensing extends into public health, where UV disinfection, infrared thermal imaging, and robotic automation have been deployed for safe, contactless COVID-19 mitigation [90]. Complementing these approaches, research on infrared thermometry highlights site-specific forehead measures that perform best indoors and wrist readings that remain stable outdoors, reinforcing the importance of context in non-contact design [91].

Further support comes from Shaik et al. [92], whose review of RPM systems emphasizes architectures designed for background functionality, leveraging edge computing, cloud integration and AI-based anomaly detection. They advocate for ambient intelligence systems that preserve privacy, reduce friction and adapt intelligently to users’ needs, supporting Pi-CON’s relevance in shaping future system requirements. A recent proof-of-concept by Baumann & Stone [93] demonstrates the practical application of the Pi-CON framework in sensor development. Their study introduces a novel, non-contact, continuous vital-sign monitoring system designed explicitly with Pi CON principles as core design requirements.

Together, these studies demonstrate a clear trajectory toward unobtrusive, ambient, and clinically relevant health monitoring systems. By integrating radar, optical, and infrared modalities with advanced AI-driven analytics, these technologies align strongly with the Pi-CON principles of passive, non-contact and continuous sensing, paving the way for scalable adoption in smart healthcare environments.

As smart healthcare matures, frameworks like Pi-CON will become increasingly important. This is not only for evaluating emerging products but also for guiding the development of systems that are clinically effective and invisible by design. Passive operation reduces fatigue, non-contact sensors preserve comfort, and continuous monitoring enables earlier detection and intervention. These principles offer a cohesive roadmap to ensure AI and IoT-enabled health systems are not only intelligent but also inclusive, accessible and integrated into daily life. Therefore, Pi-CON not only highlights the design and engagement barriers inherent in wearable technologies but also translates these insights into strategic recommendations for developing systems that are accessible, intelligent and aligned with user needs.

## 5. Discussion

### 5.1. Key Findings

This review highlights that while AI-enabled home health technologies show significant promise, their adoption is hampered by recurring barriers in usability, long-term engagement, trust and population inclusivity. Across the literature, it is evident that many devices are technologically sophisticated but fail in real-world deployment due to poor onboarding, inadequate support for diverse user needs and limited integration into daily life. Studies consistently show that user engagement drops sharply after initial use, especially in populations with lower digital literacy or physical impairments. Furthermore, AI models embedded in these tools, while increasingly accurate, often lack transparency and personalization. These issues collectively limit the ability of such technologies to deliver on their promise of scalable, home-based care.

Many studies discussed focus on short-term trials, omitting data on long-term adherence or real-world effectiveness. Additionally, many evaluations of AI-enabled tools do not clearly differentiate between performance in controlled environments versus uncontrolled, at-home settings. Regulatory gaps further compound these challenges, with many tools bypassing clinical validation by marketing themselves as wellness devices. Importantly, usability testing is often insufficient or missing entirely from development cycles.

### 5.2. Thematic Insights and Integration of the Pi-CON Framework

A central theme emerging from this review is the mismatch between technical capability and human-centered usability. This underscores the value of the Pi-CON methodology. It reframes technology evaluation around user burden: does the device require constant interaction? Does it rely on wearables or active input? Can it operate unobtrusively in daily life?

**Figure 2:**
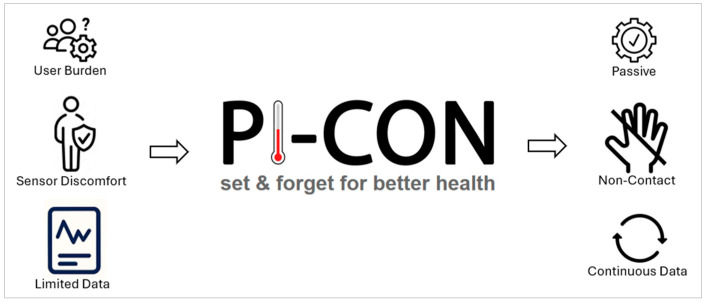
Elements of the Pi-CON methodology and its benefits.

Figure 2 illustrates the three pillars of Pi-CON (passive, non-contact and continuous), and each address a critical shortcoming identified in the literature. Passive systems reduce user burden by eliminating the need for active participation. This is essential given the high drop-off rates due to user fatigue and novelty effects. Non-contact modalities eliminate the discomfort or inconvenience associated with wearables, which are often cited as barriers by older users or those with physical limitations. Continuous monitoring ensures that data collection is not episodic or easily interrupted, allowing for richer trend analysis and more reliable health insights. The purpose of Figure 2 is to conceptually summarize the principles of Pi-CON as they relate to the broader review topic of unobtrusive health monitoring.

Equally important is the concept of ubiquity, or the “set-and-forget” nature of Pi-CON-aligned systems. Once installed, these systems integrate seamlessly into a user’s environment without demanding ongoing input. This quality aligns with the strongest predictors of long-term adherence reported in the literature: to include minimal cognitive and behavioral friction, clear data interpretation and unobtrusive design.

By re-centering evaluation around these dimensions, Pi-CON helps identify which technologies are truly suited for scalable, inclusive home health use. For example, radar-based vitals sensing or camera-driven body composition tools exemplify Pi-CON alignment, while wrist-worn devices that require regular charging and manual input often do not. This lens offers a more nuanced understanding of why many AI-powered tools fail to achieve long-term engagement despite their technical sophistication.

### 5.3. Practical Implications

The findings in this review, viewed through the Pi-CON lens, have important implications for developers, regulators and clinicians. Developers should integrate Pi-CON criteria into the product design process, emphasizing minimal user interaction, non-contact sensing and continuity of data collection. This could involve prioritizing ambient sensors, automated feedback systems, or devices that blend into users’ daily routines. For clinicians, tools that meet Pi-CON criteria may be more reliable for long-term monitoring due to higher user adherence.

Regulators may also consider adopting Pi-CON-aligned evaluation standards for Software as a Medical Device (SaMD), focusing on real-world usability and sustained engagement rather than only accuracy in lab settings. Finally, Pi-CON could serve as a benchmarking tool to guide procurement, ensuring that institutional investments support solutions likely to succeed in diverse home settings.

### 5.4. Future Research Directions

Future work should evaluate how Pi-CON-aligned tools perform across diverse populations and over extended periods. Comparative studies that test Pi-CON versus non-Pi-CON tools in terms of adherence, trust and outcomes could provide empirical support for broader adoption. In particular, researchers should focus on technologies that achieve contactless monitoring while minimizing cognitive and physical demands, such as radar-based or AI-powered camera tools. There is also a need to develop standardized metrics for evaluating passive and continuous sensing tools, ideally with interdisciplinary collaboration between clinicians, engineers and usability experts.

Finally, expanding the role of consumer smartphones that are already a cornerstone in AI-enabled health monitoring represents a promising direction for Pi-CON-aligned development. These devices contain embedded sensors that can support passive, non-contact data collection without requiring new hardware. For instance, photoplethysmography (PPG) and pulse transit time (PTT) can estimate heart rate and blood pressure via the phone’s camera and flash. Microphones and speakers can assess respiration and detect coughs, and accelerometers can be used for ballistocardiography (BCG), capturing cardiac signals through subtle body motion. When left idle on a bedside table, smartphones can unobtrusively collect rich physiological data, providing an ambient, user-friendly alternative aligned with Pi-CON’s emphasis on minimal burden, continuous monitoring and non-intrusiveness.

## 6. Conclusions

In conclusion, integrating Pi-CON into research and product development can guide the creation of AI-enabled health technologies that not only work but work for real people in real homes.

AI-enabled wearables and diagnostic tools represent a transformative shift in healthcare delivery, enabling users to engage in real-time, home-based health monitoring. However, despite technological maturity in areas such as arrhythmia detection, digital body composition analysis and mobile diagnostics, many tools struggle to sustain user engagement, demonstrate cross-population usability, and maintain user trust over time. This review shows that these challenges are deeply rooted in the friction between device design and user realities: complex onboarding processes, lack of personalization, and inconsistent integration into everyday life hinder long-term adoption.

The Pi-CON framework, emphasizing Passive, Non-contact, and Continuous monitoring, offers a practical evaluative lens to identify which technologies are poised for sustainable success in home settings. Unlike traditional frameworks centered on sensor type or platform, Pi-CON redefines success by focusing on user burden, frictionless integration, and the potential for ubiquitous deployment.

The adoption of the Pi-CON is visible in independent research that has applied or referenced the method across multiple domains of digital health [94]. Recent investigations in remote patient monitoring [95], as well as telehealth and telerehabilitation frameworks [96,97], have pointed to the feasibility of continuous, non-contact and passive sensing in real-world clinical and home settings. In parallel, design-oriented studies on wearable and ambient health tracking devices [98] and infrastructure-oriented research on RFID and smart sensing networks [99] further highlight how Pi-CON aligns with emerging requirements for unobtrusive, user-centered monitoring. Similar frameworks, such as camera-based vital sign monitoring, radar-based home sensing, and ambient IoT networks also illustrate that the field is broadly moving toward ubiquitous, low-burden monitoring. In addition, recent work on AI-driven, multimodal models that integrate camera, radar, thermal, and ambient sensors to simultaneously track multiple vital signs without contact [100,101] demonstrate that the field is advancing toward comprehensive, hands-free health monitoring systems in virtual care.

As the healthcare industry continues to shift care away from centralized facilities and into homes, Pi-CON provides not only a benchmark for evaluating devices but also a design philosophy that centers on ease, dignity, and accessibility.

To accelerate meaningful progress, developers, regulators and researchers should incorporate Pi-CON principles from the outset. Developers should prioritize ambient, passive systems that reduce user workload. Regulators and funding agencies should demand usability metrics alongside accuracy benchmarks. Researchers should test new AI-enabled solutions in diverse populations and across long-term, real-world conditions.

Ultimately, achieving the promise of smart healthcare at home will require more than technical innovation. It will require systems that are empathetic to users’ needs, resilient across environments and capable of fading into the background, silently delivering insight without demanding attention. With Pi-CON as a guiding framework, we are better positioned to build and evaluate AI-enabled health technologies that are not only intelligent, but truly usable and inclusive.
